# Fasting Glucose State Determines Metabolic Response to Supplementation with Insoluble Cereal Fibre: A Secondary Analysis of the Optimal Fibre Trial (OptiFiT)

**DOI:** 10.3390/nu11102385

**Published:** 2019-10-06

**Authors:** Stefan Kabisch, Nina M. T. Meyer, Caroline Honsek, Christiana Gerbracht, Ulrike Dambeck, Margrit Kemper, Martin A. Osterhoff, Andreas L. Birkenfeld, Ayman M. Arafat, Mads F. Hjorth, Martin O. Weickert, Andreas F. H. Pfeiffer

**Affiliations:** 1 Department of Clinical Nutrition, German Institute of Human Nutrition Potsdam-Rehbrücke, Arthur-Scheunert-Allee 114-116, 14558 Nuthetal, Germany; nina.meyer@dife.de (N.M.T.M.); caroline.honsek@gmail.com (C.H.); Christiana.Gerbracht@dife.de (C.G.); ulrikedambeck@gmx.de (U.D.); margrit.kemper@dife.de (M.K.); martin@cm-osterhoff.de (M.A.O.); Ayman.Arafat@charite.de (A.M.A.); afhp@dife.de (A.F.H.P.); 2 Deutsches Zentrum für Diabetesforschung e.V., Geschäftsstelle am Helmholtz-Zentrum München, Ingolstädter Landstraße 1, 85764 Neuherberg, Germany; Andreas.Birkenfeld@uniklinikum-dresden.de; 3 Department of Endocrinology, Diabetes and Nutrition, Campus Benjamin Franklin, Charité University Medicine, Hindenburgdamm 30, 12203 Berlin, Germany; 4 Section of Metabolic Vascular Medicine, Medical Clinic III and Paul Langerhans Institute Dresden of the Helmholtz Center Munich at University Hospital and Faculty of Medicine, TU Dresden, Fetscherstraße 74, 01307 Dresden, Germany; 5 Section of Diabetes and Nutritional Sciences, Rayne Institute, Denmark Hill Campus, King’s College London, London SE5 9NT, UK; 6 Department of Nutrition, Exercise, and Sports, Faculty of Science, University of Copenhagen, 2200 Copenhagen, Denmark; madsfiil@nexs.ku.dk; 7 Warwickshire Institute for the Study of Diabetes, Endocrinology and Metabolism, The ARDEN NET Centre, ENETS CoE, University Hospitals Coventry and Warwickshire NHS Trust, Coventry CV2 2DX, UK; M.weickert@outlook.com; 8 Centre of Applied Biological & Exercise Sciences (ABES), Faculty of Health & Life Sciences, Coventry University, Coventry CV1 5FB, UK; 9 Translational & Experimental Medicine, Division of Biomedical Sciences, Warwick Medical School, University of Warwick, Coventry CV4 7AL, UK

**Keywords:** diabetes mellitus type 2, prediabetes, diabetes prevention, impaired fasting glucose, stratification, impaired glucose tolerance, insoluble dietary fibre, insulin sensitivity

## Abstract

Background: High intake of cereal fibre is associated with reduced risk for type 2 diabetes and long-term complications. Within the first long-term randomized controlled trial specifically targeting cereal fibre, the Optimal Fibre Trial (OptiFiT), intake of insoluble oat fibre was shown to significantly reduce glycaemia. Previous studies suggested that this effect might be limited to subjects with impaired fasting glucose (IFG). Aim: We stratified the OptiFiT cohort for normal and impaired fasting glucose (NFG, IFG) and conducted a secondary analysis comparing the effects of fibre supplementation between these subgroups. Methods: 180 Caucasian participants with impaired glucose tolerance (IGT) were randomized to twice-a-day fibre or placebo supplementation for 2 years (*n* = 89 and 91, respectively), while assuring double-blinded intervention. Fasting blood sampling, oral glucose tolerance test and full anthropometry were assessed annually. At baseline, out of 136 subjects completing the first year of intervention, 72 (54%) showed IFG and IGT, while 64 subjects had IGT only (labelled “NFG”). Based on these two groups, we performed a stratified per-protocol analysis of glycometabolic and secondary effects during the first year of intervention. Results: The NFG group did not show significant differences between fibre and placebo group concerning anthropometric, glycometabolic, or other biochemical parameters. Within the IFG stratum, 2-h glucose, HbA1c, and gamma-glutamyl transferase levels decreased more in the fibre group, with a significant supplement x IFG interaction effect for HbA1c. Compared to NFG subjects, IFG subjects had larger benefits from fibre supplementation with respect to fasting glucose levels. Results were robust against adjustment for weight change and sex. An ITT analysis did not reveal any differences from the per-protocol analysis. Conclusions: Although stratification resulted in relatively small subgroups, we were able to pinpoint our previous findings from the entire cohort to the IFG subgroup. Cereal fibre can beneficially affect glycemic metabolism, with most pronounced or even isolated effectiveness in subjects with impaired fasting glucose.

## 1. Introduction

The worldwide prevalence of Type 2 diabetes mellitus (T2DM) is rising quickly. T2DM accounts for a huge proportion of patients with premature death and morbidity, leading to cardiovascular disease, cancer, and other long-term complications. Although about 50% of the disorder can be explained by genetic influences, T2DM onset and progression are also widely modulated by eating behaviour, physical activity, and other factors of a healthy lifestyle. Prevention and therapy of T2DM are therefore based on these elements and several large prevention trials have demonstrated their enormous potential; total risk reduction of diabetes incidence of about 40–60% was consistently possible [1,2,3,4]. 

Besides overeating and obesity, some specific nutritional factors have been identified in epidemiological studies, such as high intake of saturated fat, absence or overconsumption of alcohol and low intake of dietary fibre [5]. These factors are usually addressed jointly in order to facilitate the most effective prevention. In Germany, the PREDIAS scheme was developed on this basis. It entails a one-year course with an intensified first phase followed by a second maintenance phase with moderate counselling intensity, including various counselling techniques, which addresses different dietary aspects [6]. 

The Optimal Fibre Trial (OptiFiT), first published in 2018, was the first large, long-term, randomized controlled trial (RCT) investigating the effects of insoluble cereal fibre. OptiFiT was conducted in subjects with a high risk of diabetes. Although cohort studies have pointed to cereal fibre already more than a decade before, there is still lack of interventional proof that nonfermentable dietary fibre from grains and legumes is in fact improving metabolic state. OptiFiT demonstrated a very small effect on HbA1c as a secondary outcome, while the primary outcome on diabetes incidence was not achieved and 2-h glucose levels only reached statistical significance in women [7]. 

Recently, several publications highlighted, that T2DM is not a homogenous metabolic disorder. Cohort studies on new-onset T2DM patients discovered several phenotype clusters, distinguishing patients with mild metabolic disturbance from subjects with higher risk of complications on the basis of very simple anthropometric or metabolic parameters. Within these clusters, glycaemic state is a major component of cluster definitions [8,9]. 

Such stratification techniques are also relevant for intervention studies, as various (pre-)diabetes subgroups may respond differently to prevention and therapy. There is evidence that fasting glucose levels affect the metabolic benefit from a diet with low glycaemic index [10]. Fibre-rich diets may explicitly improve glycaemic outcomes in subjects with impaired fasting glucose (IFG) [11].

We therefore decided to perform a stratified analysis of the Optimal Fibre Trial based on pretreatment glycaemic state. We hypothesize that subjects with IFG experience a stronger benefit from one year of fibre supplementation than subjects with normal fasting glucose.

## 2. Research Design and Methods

The recruitment, inclusion, and exclusion criteria, as well as the overall study design, have been published elsewhere [7]. The Optimal Fibre Trial recruited 180 subjects with impaired glucose tolerance (IGT) in order to investigate subjects with an increased risk of diabetes onset. Most of the subjects additionally fulfilled the definition of Metabolic Syndrome. 136 subjects completed the first year of intervention and thus formed the per-protocol dataset.

For the presented work, the cohort was stratified by baseline glycaemic state, resulting in 72 subjects with combined IFG and impaired glucose tolerance (IGT) and 64 with normal fasting glucose (NFG) and IGT. IFG was defined by capillary glucose levels above 90.0 mg/dl.

All participants started the 24 month study with the one-year lifestyle program PREDIAS, which is a structured “Treatment and Education Program for Prevention of Type 2 Diabetes” [12]. It contained group-based consultations at regular intervals and consisted of 12 individual 2 hr lessons divided into a core intervention (8 lessons in 8 weeks) and booster sessions (4 lessons throughout the following 10 months) [6]. 

We further specified our dietary approach, differing from the dietary targets of the original PREDIAS publication. We defined goals for change in diet quality that subjects were asked to fulfil. These goals were in accordance with the recommendations of the German Society for Nutrition (DGE): fat intake below 30 kcal%, intake of saturated fat below 10 kcal%, and intake of dietary fibre above 15 g/1000 kcal. We also aimed for higher levels of physical activity (240 min/week) than the original PREDIAS recommendation (150 min/week) [6]. We recommended frequent ingestion of whole-grain products, legumes, vegetables, fruits, in particular berries, milk and meat products with low fat content, soft margarines, and vegetable oils rich in unsaturated fatty acids. Pedometers and the European Physical Activity Questionnaire (EPAQ)-2 were used in order to monitor physical activity (PA) [13]. 

Our participants completed food records for four consecutive days, including one weekend day, at baseline, and every six months from that. Nutrient intake was determined using the nutrition software PRODI^®^ 5.8 (Nutri-Science, Hausach, Baden-Wuerttemberg, Germany) based on Bundeslebensmittelschlüssel 3.0 [14]. 

Parallel to the PREDIAS dietary consultation, all participants received a drinking supplement, which should be used for the entire year and for an additional year after discontinuation of PREDIAS. Thus, supplementation lasted for 24 months.

### 2.1. Dietary Supplement

Details on the supplementation procedure, measurements and laboratory parameters have been given elsewhere [7]. All participants were provided with drinking supplements containing either a purified fibre extract derived from oat hulls (“fibre”; 70 wt% cellulose, 25 wt% hemicellulose and 3–5 wt% lignin (Vitacel OF 560-30; Rettenmaier & Söhne, Holzmuehle, Germany)) or a waxy maize starch with negligible content of insoluble fibre and guar gum and isomaltulose (“placebo”). Supplements should be consumed twice-a-day after dissolving the recommended amount of drinking powder in 300 mL of water. The fibre supplement added 15 g of mainly insoluble fibre per day (7.5 g per serving) to the normal nutrition. Both supplements were similar in appearance, taste, odour, and texture. Allocation was blinded to both participants and study personnel. Supplement tins were weighed accurately before distribution and weighed again when returned after use at the main visits.

Randomisation to fibre or placebo supplementation led to the formation of the following subgroups: NFG fibre (*n* = 32), NFG placebo (*n* = 32); IFG fibre (*n* = 35), IFG placebo (*n* = 37).

### 2.2. Calculations

Areas under the curve (AUC) for oral glucose tolerance test (OGTT) responses (plasma glucose, insulin, and C-peptide) were calculated by the trapezoidal method. Insulin resistance was determined by the homeostasis model assessment (HOMA-IR) [15], the ISI_ffa_ [16], and dynamic insulin sensitivity indices, described by Belfiore, Aloulou, and Cederholm [16,17,18]. We also calculated the fatty liver index (FLI) [19].

### 2.3. Statistical Analyses

We used the Kolmogorov–Smirnov test in order to determine normal distribution of our data. Given frequent absence of normal distribution, we decide to conduct nonparametric tests all over the trial to ensure uniform representation, Mann–Whitney tests for cross-sectional comparisons and Wilcoxon tests for longitudinal comparisons. In case of significant differences between groups, a 3-way ANOVA (mixed linear model: time x diet x IFG baseline status) was conducted. Adjustments for weight change and sex were included to account for possible group differences. All data are presented as means ± standard deviation. The results were considered significantly different if *p* < 0.05. All statistical analyses were performed using SPSS for Windows program version 22.0 (SPSS Inc, Chicago, IL, USA). 

## 3. Results

Sex distribution differed between both NFG subgroups. Besides that, there were no differences in baseline characteristics between the respective fibre groups and their placebo counterparts for the NFG (Table 1) and IFG strata (Table 2). IFG subjects were characterized by more strongly deranged levels of fasting glucose, body weight, measures of fasting and dynamic insulin resistance (HOMA-IR and Belfiore Index), fatty liver index, uric acid, and inflammatory parameters at baseline.

Intake of calories, macronutrients, and dietary fibre from conventional food did not differ between the four subgroups at baseline after one year. Overall, the dietary intake of calories and total fat decreased in all four subgroups, while alcohol intake decreased significantly in all but the IFG placebo group, and protein intake decreased only in the NFG fibre group. However, there were no differences between the four groups concerning dietary changes. Neither did PA change in any of the four subgroups, nor were there differences between the changes of PA of any group (Table 3).

Average fibre intake by supplementation was 1 ± 0 g in the placebo groups and 13 ± 2 g in the fibre groups.

The two NFG groups did not differ with regard to the change of anthropometric or metabolic parameters over the one-year intervention period. The NFG fibre group exclusively experienced a significant decline in hip circumference, blood pressure, and an increase in fasting glucose. The NFG placebo group, but not its fibre counterpart, showed a significant decrease in waist-to-hip ratio (WHR) and quantitative insulin sensitivity check index (QUICKI). Within both NFG subgroups, significant improvements were detected for body weight, waist circumference, 2-h glucose levels, ISI_ffa_, Belfiore index, hepatic insulin clearance (HIC), and fatty liver index (FLI) (Table 4).

Within the IFG stratum, 2-h glucose, HbA1c, and gamma-glutamyl transferase (GGT) decreased significantly more in the fibre group. These differences also remained significant after adjustment for weight change and sex. 2-h glucose, leukocyte count, C-reactive protein (CRP), and FLI improved only in the fibre group. HbA1c increased significantly in the placebo group, while there was no change in the fibre group. Significant within-group effects for body weight, waist circumference, fasting glucose, fasting insulin, HOMA_IR_, QUICKI, Belfiore index, and HIC were seen in both intervention groups of the IFG stratum (Table 4).

When comparing NFG and IFG subgroups within the fibre intervention, IFG had a significantly larger benefit with respect to 2-h glucose levels. Comparison of NFG and IFG subgroups within the placebo arm revealed a significantly stronger benefit for IFG patients with respect to fasting glucose, and a significantly stronger improvement of 2-h glucose and HbA1c in the NFG group. The interaction effect (time x supplement x IFG status; adjusted for weight change and sex) was significant for HbA1c (*p* = 0.002), but not for fasting glucose, 2-h glucose and GGT (Table 4).

There was no difference in drop-out rates: IFG placebo (13/50 = 26%), IFG fibre (13/48 = 27%), NFG placebo (9/41 = 22%), NFG fibre (9/41 = 22%). Intention-to-treat analysis did not lead to different results.

## 4. Discussion

As hypothesized, participants with impaired fasting glucose had a stronger benefit from fibre supplementation than NFG subjects. Only in IFG subjects, fibre supplementation resulted in significantly improved levels of 2-h glucose, GGT, and HbA1c compared to placebo. This led to a significant interaction effect for HbA1c, favouring fibre in IFG subjects. IFG subjects had stronger reductions of fasting glucose irrespective of supplement allocation in comparison with NFG counterparts. Therefore, we did neither find a statistical difference between fibre and placebo for fasting glucose, nor a significant interaction based on fasting glucose state and supplement group. 

The Optimal Fibre Trial was the first RCT to investigate long-term effects of cereal insoluble fibre (mainly cellulose and hemicellulose) and focused on diabetes onset. The reported effects in the original publication were small in magnitude and partially failed to reach statistical significance. In this post hoc analysis, we now provide evidence that the borderline effect of the fibre supplementation observed in the entire sample was in fact driven by a well-defined subgroup effect. The analysed cohort was recruited only by the presence of IGT, which is more common in women. IGT is also more strongly linked to impaired insulin secretion and impaired postprandial insulin action rather than fasting (hepatic) insulin sensitivity. Those imbalances in the cohort structure may somewhat explain the small effect [7].

In our current paper, we tried to pinpoint a potential metabolic benefit of cereal fibre to a more defined subtype of prediabetes. IFG and fasting insulin resistance rather than IGT and dynamic insulin resistance are strongly associated with hepatic insulin resistance and nonalcoholic fatty liver disease (NAFLD) [20,21,22]. Hepatic insulin resistance can only be assessed specifically by conducting isotope-based euglycaemic-hyperinsulinemic clamps. Such data, however, was not available in OptiFiT. Also, there was only a small subgroup of patients, whose liver fat content was measured by magnetic-resonance spectroscopy (MR-S). Therefore, neither hepatic insulin resistance nor NAFLD state could be used to stratify the cohort. Stratification by IFG state led to subgroups of comparable size and, most notably, without widespread statistical differences in anthropometric or metabolic parameters at baseline between the respective fibre and placebo arms. As expected, IFG subjects were characterized not only by higher fasting glucose levels but also by higher body weight, measures of fasting and dynamic insulin resistance (HOMA-IR and Belfiore Index), fatty liver index, uric acid levels, and inflammatory parameters at baseline.

The first main finding of this stratified analysis is a replication of the major effects of the primary OptiFiT publication. Within the IFG subset, fibre supplementation led to a significantly stronger improvement of 2-h glucose levels and HbA1c. These effects were robust after adjustment for weight change and sex despite a resulting considerably lower statistical power; for HbA1c, a significant interaction effect was found. Thus, the fibre effect goes beyond mere consolidation of prediabetic state by actually lowering glucose levels and promoting prediabetes remission. As a nonglycaemic outcome, fibre and placebo subgroups in the IFG class also differed in their change of GGT levels. This might indicate a possible mode of action. Up to now, the specific processes underlying metabolic improvements by cereal fibre intake are not fully understood. Neither incretins nor fermentation have so far been consistently identified as crucial factors in most studies [23,24]. However, baseline gut microbiota may be a determinant [25]. Data from the ProFiMet Trial (Protein, Fibre, Metabolic Syndrome) suggest that insoluble fibre intake promotes the faecal excretion of BCAAs, thereby reducing their serum levels. BCAAs are activators of the mTOR pathway and may impair insulin sensitivity [26]. However, ProFiMet did not report an effect on liver fat content through fibre intake. 

In the NFG subset of OptiFiT no differences were observed between the fibre and placebo interventions. Both interventions were accommodated by weight loss and concomitant improvement of insulin sensitivity and fatty liver index. 

When comparing the fibre subgroups, IFG subjects showed a larger improvement in fasting glucose. As the same observation was done when comparing the placebo subgroups, this is likely to be explained by the difference in baseline levels and thereby partly due to regression towards the mean. 

Additionally, NFG placebo subjects showed a larger improvement in postprandial glucose and HbA1c than IFG placebo subjects, which was not explained by different baseline values. These differential changes may possibly be caused by metabolic factors, such as insulin resistance or inflammatory state, which were indeed significantly higher in the IFG group. From that perspective, IFG placebo subjects would appear to be “resistant” to the lifestyle treatment (PREDIAS) and lacked an active supplement. NFG subjects did not benefit from fibre supplementation but at least improved by participation in the PREDIAS intervention. Fibre supplementation was apparently capable to provide IFG subjects with a benefit in HbA1c and postprandial glucose similar to both NFG subsets, although the effect of the PREDIAS intervention was rather limited in the IFG subset.

We therefore see replicated and pronounced evidence for the glycometabolic benefit of cereal fibre in subjects with prediabetes. The specific effect of insoluble fibre rather than soluble fibre needs to be investigated more thoroughly [27]. We propose that a possible way of action may be linked to the presence of NAFLD, which is often associated with IFG and, in combination with each other, leads to a substantially higher risk for diabetes onset [28]. Previous studies have already indicated that IFG state may facilitate the beneficial effects of cereal fibre [10,11]. Such interaction effects between metabolic state and fibre-driven metabolic improvements have also been reported for inulin [29]. 

Compared to previous RCTs on dietary fibre, OptiFiT provides several strengths: larger overall power, and extensive metabolic phenotyping, as well as the inclusion of both female and male subjects. In addition to that, we argue that in comparison to many previous mechanistic studies on cereal fibre, several potential confounders can be ruled out in our analysis. Firstly, all four subgroups received the same prediabetes prevention program. Food records of all four groups indicate that the PREDIAS program enabled all subgroups to decrease saturated fat intake, leading to caloric restriction and weight loss. Reduced intake of saturated fats may explain reduced levels of inflammatory parameters, blood pressure, and insulin resistance even in the absence of weight loss [30,31,32,33]. Furthermore, baseline and change in PA between the four subgroups did not differ, and we assume that this element of lifestyle change did not explain differences between the groups. Also, there was no difference in weight loss between the respective subgroups. By post-study questionnaires we were able to systematically assess side effects of the supplementation. We did not find group differences in the frequency of bloating or any other specific gastrointestinal side effect.

Weight loss was similar in all four groups, and differences in metabolic outcomes between the groups remained significant after adjustment for weight change and sex. 

Previous trials on insoluble cereal fibre focused on fibre-rich food, but many studies reported poor compliance to the advice for fibre-rich diet [34]. By supplementation, we eliminated this problem and determined high compliance in most of our subjects by drug accounting and post-study questionnaires. Furthermore, supplementation assured that the metabolic effects can be attributed to dietary fibre only and not the whole food matrix, glycaemic index of digestible carbohydrates, or vitamins and minerals from whole grain products. 

Assessing compliance is always both crucial and hard, especially in nutritional studies. By using four-day food records and continuous drug accounting, we were able to monitor compliance to dietary consultation and adherence to supplementation. We did not find evidence for extensive underreporting. At any rate, biomarkers for further evaluation of dietary compliance are not available.

Still, our subgroup analysis of OptiFiT itself has to be evaluated under consideration of some limitations. There was no preselected definition for specific subgroups, leaving the chance to random post hoc significance. However, our results replicated the findings of the original analysis quite consistently.

OptiFiT experienced a one-year drop-out rate of 24%, which is comparable to other lifestyle intervention studies [1,2,3,4]. Drop-outs due to incident T2DM mainly occurred after the one-year visit, therefore not affecting the results of this analysis. 

We are aware that OptiFiT lacks an additional randomized control group without any kind of intervention. This option was intentionally excluded for statistical and ethical reasons. 

Further studies should also include an assessment of the gut microbiome. We did not find evidence for increased GI side effects, but even low-fermentable fibre as in our supplement might influence gut bacteria in some way.

## 5. Conclusions

In conclusion, we demonstrate that supplementation with insoluble cereal fibre leads to a glycometabolic improvement (2-h glucose and HbA1c) in subjects with IFG but not with NFG. IFG subjects, furthermore, show a significantly larger reduction in GGT levels compared to NFG subjects, which might highlight a mechanistic pathway for the glycometabolic effects involving NAFLD. The results indicate that NFG subjects mainly benefit from the PREDIAS program, while IFG subjects require fibre supplementation to achieve the same metabolic benefit as NFG subjects. We provide further evidence that insoluble dietary fibre is a relevant nutritional target for future clinical trials.

## Figures and Tables

**Table 1:** Characteristics of participants at study entry (normal fasting glucose (NFG) subgroups only).

	NFG Fibre	NFG Placebo
Sex w/m	24/8 (75% female)	16/16 (50% female) *
Age (years)	61.3 ± 9.5	60.3 ± 10.5
BMI (kg/m²)	31.3 ± 5.7	32.1 ± 5.3
Weight (kg)	84.7 ± 17.8	89.4 ± 17.0
Waist circumference (cm)	100.7 ± 15.4	105.2 ± 12.2
Hip circumference (cm)	111.8 ± 12.8	111.8 ± 12.8
Waist-to-hip-ratio (WHR)	0.90 ± 0.08	0.94 ± 0.10
BIA – Body fat (%)	36.8 ± 7.4	36.8 ± 7.8
RR syst. (mmHg)	142 ± 19	141 ± 13
Fasting glucose (mg/dl)	81.7 ± 6.4	82.8 ± 5.5
2-h glucose (mg/dl)	153.4 ± 13.3	156.3 ± 15.1
HbA_1c_ (%)	5.5 ± 0.4	5.5 ± 0.3
Fasting Insulin (mU/l)	8.0 ± 4.7	8.7 ± 3.5
Fasting C-Peptide (µg/l)	1.4 ± 0.5	1.4 ± 0.5
HOMA-IR	2.0 ± 1.3	2.2 ± 0.9
QUICKI	0.36 ± 0.04	0.35 ± 0.03
ISI_ffa_	0.87 ± 0.34	0.83 ± 0.24
Belfiore	0.68 ± 0.27	0.69 ± 0.27
HIC_c-peptide_ (mU/µg)	4.8 ± 1.8	4.8 ± 1.4
HDL cholesterol (mmol/l)	1.3 ± 0.3	1.3 ± 0.3
LDL cholesterol (mmol/l)	3.8 ± 0.8	3.5 ± 0.6
CRP (mg/l)	3.6 ± 5.4	3.1 ± 3.2
Leukocyte count (Gpt/l)	5.61 ± 1.72	4.96 ± 0.91
Uric acid (µmol/l)	320 ± 58	347 ± 79
GGT (U/l)	32 ± 42	37 ± 36
Fatty liver index (FLI)	62 ± 29	68 ± 29

Characteristics of NFG participants at study entry, *Significant difference between the groups, * *p* < 0.05.

**Table 2:** Characteristics of participants at study entry (impaired fasting glucose (IFG) subgroups only).

	IFG Fibre	IFG Placebo
Sex	24 / 11 (69% female)	20 / 17 (54% female)
Age (years)	58.9 ± 9.1	59.7 ± 8.1
BMI (kg/m²)	32.2 ± 4.6	34.5 ± 7.4
Weight (kg)	90.3 ± 12.7	97.9 ± 23.1
Waist circumference (cm)	104.0 ± 9.4	108.2 ± 16.4
Hip circumference (cm)	111.3 ± 10.1	116.8 ± 14.0
Waist-to-hip-ratio (WHR)	0.94 ± 0.08	0.93 ± 0.09
BIA – Body fat (%)	37.0 ± 9.2	34.8 ± 8.4
RR syst. (mmHg)	137 ± 16	142 ± 19
Fasting glucose (mg/dl)	97.7 ± 6.6	99.0 ± 6.4
2-h glucose (mg/dl)	161.8 ± 18.0	165.5 ± 20.8
HbA_1c_ (%)	5.7 ± 0.4	5.7 ± 0.4
Fasting Insulin (mU/l)	9.9 ± 4.2	10.8 ± 6.5
Fasting C-Peptide (µg/l)	1.9 ± 0.8	1.8 ± 0.8
HOMA-IR	2.7 ± 1.2	3.1 ± 2.1
QUICKI	0.33 ± 0.02	0.34 ± 0.04
ISI_ffa_	0.85 ± 0.24	0.81 ± 0.34
Belfiore	0.63 ± 0.27	0.64 ± 0.31
HIC_c-peptide_ (mU/µg)	5.0 ± 1.6	5.2 ± 2.4
HDL cholesterol (mmol/l)	1.2 ± 0.2	1.2 ± 0.3
LDL cholesterol (mmol/l)	3.7 ± 1.0	3.5 ± 0.8
CRP (mg/l)	5.2 ± 4.1	3.1 ± 4.0
Leukocyte count (Gpt/l)	5.87 ± 1.29	5.82 ± 1.63
Uric acid (µmol/l)	358 ± 84	351 ± 85
GGT (U/l)	37 ± 26	29 ± 22
Fatty liver index (FLI)	74 ± 21	71 ± 27

Characteristics of IFG participants at study entry, No significants differences between the groups.

**Table 3:** Lifestyle habits at baseline and after one year of intervention.

	NFG Fibre (Baseline)	NFG Placebo (Baseline)	IFG Fibre (Baseline)	IFG Placebo (Baseline)	NFG Fibre (12 Months)	NFG Placebo (12 Months)	IFG Fibre (12 Months)	IFG Placebo (12 Months)
Food intake								
Total energy intake (kcal/day)	1970 ± 386	2054 ± 626	2118 ± 532	1969 ± 541	1698 ± 414 *	1888 ± 469 *	1772 ± 360 *	1933 ± 607 *
Fat intake (g/day)	75 ± 22	78 ± 26	85 ± 26	79 ± 31	64 ± 24 *	70 ± 26 *	66 ± 21*	73 ± 32 *
Protein intake (g/day)	78 ± 18	82 ± 23	85 ± 26	84 ± 22	66 ± 17 **	81 ± 21	75 ± 21	81 ± 23
Carbohydrate intake (g/day)	226 ± 52	231 ± 82	227 ± 63	214 ± 57	205 ± 45	216 ± 47	201 ± 44	220 ± 63
Total dietary fibre intake (g/day)	23 ± 6	25 ± 9	23 ± 8	22 ± 5	23 ± 6	24 ± 10	22 ± 8	24 ± 8
Insoluble	15 ± 4	16 ± 6	15 ± 5	15 ± 4	15 ± 5	16 ± 5	14 ± 5	15 ± 5
Soluble	7 ± 2	8 ± 3	7 ± 2	7 ± 2	7 ± 2	8 ± 3	7 ± 2	7 ± 2
Alcohol (g/day)	8 ± 16	11 ± 15	12 ± 15	6 ± 12	3 ± 4 *	7 ± 8 *	8 ± 10 *	7 ± 13
Physical activity								
Steps per day	7052 ± 2924	6045 ± 2472	6382 ± 3063	6482 ± 2711	7775 ± 3348	5805 ± 2601	7185 ± 3215	7538 ± 3795
Energy expenditure by steps	499 ± 218	400 ± 160	415 ± 243	461 ± 278	534 ± 247	381 ± 188	435 ± 160	508 ± 239

Changes in lifestyle habits during intervention; supplement intake is not covered by this assessment. Nutrient intakes were calculated from four-day food records. Physical activity was derived from one-week assessments with pedometers. Data are means (SD). * Significant change within the groups, * *p* < 0.05, ** *p* < 0.001.

**Table 4:** Changes in anthropometric and metabolic parameters.

	NFG Fibre	NFG Placebo	IFG Fibre	IFG Placebo	NFG: Fibre vs. Placebo	IFG: Fibre vs. Placebo	Placebo: NFG vs. IFG	Fibre: NFG vs. IFG
Weight (kg)	−3.2 ± 5.3 **	−3.1 ± 5.5 **	−2.2 ± 3.8 ***	−3.1 ± 5.9 **	n.s.	n.s.	n.s.	n.s.
Waist circumference (cm)	−2.9 ± 5.8 **	−3.5 ± 7.4 *	−2.9 ± 4.5 ***	−3.1 ± 6.0 **	n.s.	n.s.	n.s.	n.s.
Hip circumference (cm)	−3.2 ± 4.0 ***	−1.3 ± 4.3	−1.5 ± 4.7	−3.8 ± 5.9 **	n.s.	n.s.	n.s.	n.s.
WHR	0.00 ± 0.04	−0.02 ± 0.05 *	−0.01 ± 0.04	0.00 ± 0.05	n.s.	n.s.	n.s.	n.s.
BIA – Body fat (%)	−0.4 ± 5.1	−2.2 ± 5.8	−0.6 ± 4.3	−0.2 ± 3.1	n.s.	n.s.	n.s.	n.s.
RR syst. (mmHg)	−7 ± 16 *	−2 ± 15	2 ± 16	−2 ± 19	n.s.	n.s.	n.s.	n.s.
Fasting glucose (mg/dl)	4.3 ± 9.8 *	2.7 ± 7.7	−7.6 ± 8.8 ***	−4.7 ± 7.6 **	n.s.	n.s.	† < 0.001	† < 0.001
2-h glucose (mg/dl)	−9.5 ± 24.1 *	−11.6 ± 23.8 **	−12.7 ± 33.3 **	1.8 ± 34.9	n.s.	† < 0.05	† < 0.05	n.s.
HbA_1c_ (%)	0.1 ± 0.5	−0.0 ± 0.4	−0.1 ± 0.4	0.2 ± 0.6 *	n.s.	† < 0.01	† < 0.05	n.s.
Fasting Insulin (mU/l)	−1.2 ± 3.2	−0.4 ± 4.3	−1.8 ± 3.6 **	1.8 ± 5.2 *	n.s.	n.s.	n.s.	n.s.
Fasting C-Peptide (µg/l)	0.1 ± 1.0	0.4 ± 1.3	0.1 ± 1.4	0.4 ± 1.6	n.s.	n.s.	n.s.	n.s.
HOMA-IR	−0.3 ± 0.9	−0.1 ± 1.2	−0.6 ± 1.2 **	−0.6 ± 1.5 *	n.s.	n.s.	n.s.	n.s.
QUICKI	0.00 ± 0.03	0.01 ± 0.03 *	0.01 ± 0.02 **	0.01 ± 0.03 *	n.s.	n.s.	n.s.	n.s.
ISI_ffa_	0.10 ± 0.27 *	0.10 ± 0.25 *	0.04 ± 0.28	0.09 ± 0.38	n.s.	n.s.	n.s.	n.s.
Belfiore	0.12 ± 0.30 *	0.11 ± 0.27 *	0.14 ± 0.27 **	0.16 ± 0.24 **	n.s.	n.s.	n.s.	n.s.
HIC_c-peptide_ (mU/µg)	1.0 ± 2.2 *	1.2 ± 2.3 **	1.3 ± 2.6 **	1.9 ± 2.5 ***	n.s.	n.s.	n.s.	n.s.
HDL cholesterol (mmol/l)	−0.0 ± 0.2	−0.0 ± 0.2	0.0 ± 0.1	0.0 ± 0.3	n.s.	n.s.	n.s.	n.s.
LDL cholesterol (mmol/l)	−0.1 ± 0.5	−0.1 ± 0.8	−0.1 ± 1.0	−0.2 ± 0.9	n.s.	n.s.	n.s.	n.s.
CRP (mg/l)	−0.5 ± 4.2	−0.6 ± 2.6	−1.8 ± 3.3 **	−0.5 ± 2.8	n.s.	n.s.	n.s.	n.s.
Leukocyte count (Gpt/l)	−0.33 ± 1.41	0.19 ± 1.03	−0.65 ± 1.14 *	−0.07 ± 1.04	n.s.	n.s.	n.s.	n.s.
Uric acid (µmol/l)	−7 ± 43	−11 ± 61	−14 ± 69	−6 ± 55	n.s.	n.s.	n.s.	n.s.
GGT (U/l)	−8 ± 40	−4 ± 20	−5 ± 14	6 ± 24	n.s.	† < 0.05	n.s.	n.s.
Fatty liver index (FLI)	−8 ± 17 *	−7 ± 13 **	−5 ± 12 *	−2 ± 13	n.s.	n.s.	n.s.	n.s.

Outcomes during intervention; Mean ± SD. * Significant difference within the groups; * *p* < 0.05, ** *p* < 0.01, *** *p* < 0.001.; † Significant difference between groups; n.s. = not significant.

## Abbreviations

AA: amino acid; ALAT: alanine-amino transferase; ASAT: aspartate-amino transferase; AUC: area under the curve; BCAA: branched-chain amino acids; BIA: bioelectric impedance analysis; CRP: c-reactive protein; EPAQ: European Physical; Activity Questionnaire; FLI: Fatty Liver Index; GGT: gamma-glutamyl transferase; HbA1c: Glycated hemoglobin; HDL: high-density lipoprotein; HIC: hepatic insulin clearance; HOMA-IR: Homeostasis model assessment insulin resistance index; IFG: impaired fasting glucose; IGT: impaired glucose tolerance; ISI_ffa_: Insulin sensitivity index of blood free fatty acids; LDL: low-density lipoprotein; MR-S: magnetic-resonance spectroscopy; mTOR: mechanistic Target of Rapamycin; NAFLD: Non-alcoholic fatty liver disease; NFG: Normal Fasting Glucose; OptiFiT: Optimal Fibre; Trial for diabetes prevention; OGTT: oral glucose tolerance test; PA: physical activity; PREDIAS: Prevention of Diabetes Self-Management; ProFiMet: Protein, Fibre, Metabolic Syndrome; QUICKI: Quantitative insulin sensitivity check index; Sg: glucose effectiveness; T2DM: type 2 diabetes mellitus
